# Cobalt Oxide Nanoparticles: Behavior towards Intact and Impaired Human Skin and Keratinocytes Toxicity

**DOI:** 10.3390/ijerph120708263

**Published:** 2015-07-17

**Authors:** Marcella Mauro, Matteo Crosera, Marco Pelin, Chiara Florio, Francesca Bellomo, Gianpiero Adami, Piero Apostoli, Giuseppe De Palma, Massimo Bovenzi, Marco Campanini, Francesca Larese Filon

**Affiliations:** 1 Clinical Unit of Occupational Medicine, Department of Medical Sciences, University of Trieste, Via della Pietà, Trieste 19-34129, Italy; E-Mails: marcella.mauro82@gmail.com (M.M.); crosera.matteo@gmail.com (M.C.); bellomo.francesca@gmail.com (F.B.); bovenzi@units.it (M.B.); 2 Department of Chemical and Pharmaceutical Sciences, University of Trieste, Via Giorgeri 2, Trieste 1-34127, Italy; E-Mail: gadami@units.it; 3 Department of Life Sciences, University of Trieste, Via L. Giorgeri 7/9, Trieste 34127, Italy; E-Mails: mpelin@units.it (M.P.); florio@units.it (C.F.); 4 Dipartimento di Specialità Medico Chirurgiche, Scienze Radiologiche, Sanità Pubblica, University of Brescia, Piazza del Mercato, Brescia 15-25121, Italy; E-Mails: apostoli@med.unibs.it (P.A.); giuseppe.depalma@unibs.it (G.D.P.); 5 IMEM-CNR Institute, Parco Area delle Scienze 37/A, Parma 43124, Italy; E-Mail: marco.campanini@imem.cnr.it

**Keywords:** cobalt oxide, nanoparticles, *in vitro*, human skin absorption, keratinocytes toxicity

## Abstract

Skin absorption and toxicity on keratinocytes of cobalt oxide nanoparticles (Co_3_O_4_NPs) have been investigated. Co_3_O_4_NPs are commonly used in industrial products and biomedicine. There is evidence that these nanoparticles can cause membrane damage and genotoxicity *in vitro*, but no data are available on their skin absorption and cytotoxicity on keratinocytes. Two independent 24 h *in vitro* experiments were performed using Franz diffusion cells, using intact (experiment 1) and needle-abraded human skin (experiment 2). Co_3_O_4_NPs at a concentration of 1000 mg/L in physiological solution were used as donor phase. Cobalt content was evaluated by Inductively Coupled–Mass Spectroscopy. Co permeation through the skin was demonstrated after 24 h only when damaged skin protocol was used (57 ± 38 ng·cm^−2^), while no significant differences were shown between blank cells (0.92 ± 0.03 ng cm^−2^) and those with intact skin (1.08 ± 0.20 ng·cm^−2^). To further investigate Co_3_O_4_NPs toxicity, human-derived HaCaT keratinocytes were exposed to Co_3_O_4_NPs and cytotoxicity evaluated by MTT, Alamarblue^®^ and propidium iodide (PI) uptake assays. The results indicate that a long exposure time (*i.e.*, seven days) was necessary to induce a concentration-dependent cell viability reduction (EC_50_ values: 1.3 × 10^−4^ M, 95% CL = 0.8–1.9 × 10^−4^ M, MTT essay; 3.7 × 10^−5^ M, 95% CI = 2.2–6.1 × 10^−5^ M, AlamarBlue^®^ assay) that seems to be associated to necrotic events (EC_50_ value: 1.3 × 10^−4^ M, 95% CL = 0.9–1.9 × 10^−4^ M, PI assay). This study demonstrated that Co_3_O_4_NPs can penetrate only damaged skin and is cytotoxic for HaCat cells after long term exposure.

## 1. Introduction

The use of nanoparticles (NPs) has grown in the last decades in many fields of every day life, and imposes to the scientific community to take into account their toxicological potential. In fact, NPs may have an unpredictable impact on human health, since traditional toxicological knowledge, based on data derived from materials in their bulk form, is not applicable in the nano size range. One of the crucial aspect is NPs penetration into the body and skin can be a crucial route of entry due to skin contact and skin contamination that are very common in working conditions, where risk perception of the “skin route” is very low. Moreover, to protect workers from inhalation exposure, more NPs are produced as suspension decreasing inhalation risk but increasing potentially skin absorption.

Magnetic nanoparticles have been proposed in many biomedical applications, such as cancer diagnosis [1], radioactive vectors in cancer therapy [2], and as drug delivery systems [3]. CoO and Co_3_O_4_ are two important forms among the various cobalt oxides based on their distinctive structural features and properties [4] and it has been demonstrated that these transition metal oxides, when falling in the nanosized regime, have even more attractive applications such as, e.g. heterogeneous catalysts, gas sensors, lithium ion batteries, electrochromic devices, solar energy absorbers, ceramic pigments and optical devices, *etc.* [5,6,7,8,9,10,11]. Actually, these NPs are used as contrast agents in magnetic resonance [12], as drug delivery system [13] and as adjuvants for use in human vaccination too, especially when both lymphocytes Th1 and Th2 responses are needed to clear pathogens [14]. On the other hand, some studies demonstrated the induction of membrane damage and genotoxicity in HepG2 cells through ROS and oxidative stress due to these NPs [15]. Cobalt oxide NPs are graded as harmful to humans and dangerous for the environment, but experimental data are lacking. Concerns arise because Cobalt is also a skin sensitizer [16] and a previous study of our group demonstrated that skin exposure to 80 nm CoNPs can lead to skin permeation of this metal [17]. There are no data on cobalt oxide nanoparticles behavior through skin barrier. There is the need to study if Co_3_O_4_NPs can release ions in physiological condition, if they can penetrate and permeate the skin and to understand whether skin permeation differs between metal and metal oxides NPs species.

The aim of this study was to evaluate Co_3_O_4_NPs human skin absorption, since consumers and workers exposure may increase in the next few years. We used the experience and the protocol employed during the European project EDETOX (Evaluations and predictions of DErmal absorption of TOXic chemicals), a three-year research program (2001–2004) funded by European Union [18] and already used to test the skin permeation of other metal nanoparticles such as silver, gold and cobalt [17,19,20].

## 2. Materials and Methods

### 2.1. Chemicals

All chemicals were analytical grade. Urea, sodium chloride, sodium hydrogenphosphate, potassium dihydrogenphosphate, were purchased from Carlo Erba (Milan, Italy); lactic acid (90% v/v) was bought from Acros Organics (Geel, Belgium); nitric acid (69.5% v/v), hydrogen peroxide (30% v/v), ammonium hydroxide (25% w/v) from Sigma Aldrich (Milan, Italy). Water reagent grade was produced with a Millipore purification pack system (milliQ water).

The commercially available cobalt (II,III) oxide (<50 nm) nanopowder was provided with physico-chemical characterization by Sigma (St. Louis, MO, USA).

### 2.2. Nanoparticles Characterization

The Co_3_O_4_NPs have been visualized by Transmission Electron Microscopy (TEM) using a 200 kV analytical JEM 2200-FS (JEOL Inc., Peabody, MA, USA), once they were dispersed in synthetic sweat and at the end of the experiments (after the 24 h exposure time) to visualize the dimensions of the NPs and the aggregation state of the donor phase.

In addition, since the behavior and the aggregation state of the NPs depends strongly on the surface charge of the NPs and the ionic strength of the suspension, further characterization using both Dynamic Light Scattering (DLS) and Z-potential techniques have been carried out. The measurements have been performed using the 90 Plus PALS instrument (Brookhaven Instruments Corporation, Holtsville, NY, USA).

### 2.3. Nanoparticles Dissolution

In order to evaluate the ions release from the NPs once they were put in synthetic sweat, 4 mL of the donor phase (described in *in vitro* diffusion system paragraph) have been ultrafiltered using the Amicon Ultra-4 centrifugal filters (10K MWCO) supplied by Millipore Corporation, Billerica, MA 01821 USA. The ultrafiltration has been performed in centrifuge at 5000 rpm for 30 min in order to remove the Co_3_O_4_NPs, but not cobalt ions, from the solution. The solution has been analyzed by ICP–AES (Inductively Coupled Plasma–Atomic Emission Spectroscopy) to quantify the cobalt concentration. The ultrafiltration has been repeated on three different aliquots at the beginning of the permeation experiments, and on other three aliquots at the end of the 24-h and 7-day exposure times.

### 2.4. Preparation of Skin Membranes

Human abdominal full thickness skin was obtained as surgical waste from 2 patients aged 45–65 years after obtaining ethical committee approval. After the skin excision, subcutaneous fat was removed with a scalpel blade and hair was shaved from the epidermal layer, then skin samples were stored at −25 °C for a period up to, but not exceeding, 4 months. It has been demonstrated that this procedure does not damage skin barrier properties. At the day of the experiment skin samples have been defrost in physiological solution at room temperature for a 30 min period and then 4 × 4 cm^2^ pieces were cut from each skin specimen and mounted separately on the diffusion cells. Thickness of the membranes were <1mm. Damaged skin samples were obtained using a needle-abrasion technique described elsewhere [21]. Skin integrity was tested before and after each experiment using electrical conductibility by means of a conductometer (Metrohm, 660, Metrohm AG Oberdorfstr. 68 CH-9100 Herisau) operating at 300 Hz and connected to two stainless steel electrodes [22]. The conductibility data in μS were converted into KΩ cm^−2^. Cells with a resistance lower than 3.95 ± 0.27 KΩ·cm^−2^ were considered to be damaged and rejected, as suggested by Davies *et al.* [23].

### 2.5. In Vitro Diffusion System

Percutaneous absorption studies were performed using static diffusion cells following the Franz method [24]. The receptor compartment had a mean volume of 14.0 mL and was maintained at 32 °C by means of circulation of thermostated water in the jacket surrounding the cell. This temperature value was chosen in order to reproduce the hand physiological temperature at normal conditions. The physiological solution used as the receptor phase was prepared by dissolving 2.38 g of Na_2_HP0_4_, 0.19 g of KH_2_PO_4_ and 9 g of NaCl into 1 L of milliQ water (final pH 7.35). The synthetic sweat solution used as donor fluid consisted in 0.5% sodium chloride, 0.1% urea and 0.1% lactic acid in milliQ water; pH 4.5 was adjusted with ammonia.

The concentration of the salt in the receptor fluid was approximately the same that can be found in blood. The physiological solution used as receiving phase was continuously stirred using a Teflon coated magnetic stirrer (made in UK, distributed by VWR International, Milan, Italy). Each piece of skin was clamped between the donor and the receptor compartment; the mean exposed skin area was 3.29 cm^2^ and the average membranes thickness was 1 mm. Two different experiments were conducted using intact (exp. 1) and damaged skin (exp. 2) as described below:

#### 2.5.1. Experiment 1

The donor phase has been prepared just before the experiment using a sonicated suspension of Co_3_O_4_NPs at a concentration of 1000 mg/L dispersed in synthetic sweat at pH 4.5, to reproduce *in vivo* condition. The Co_3_O_4_ concentration in the donor phase was confirmed by Inductively Coupled Plasma–Atomic Emission Spectroscopy (ICP-AES) analysis prior to the test. At time 0, the exposure chambers of 6 Franz diffusion cells were mounted with intact skin samples and filled with 2.5 mL of the donor suspension (606 μg·cm^−2^) to ensure an infinite dose. The experiment was run for 24 h, and during this period 1.5 mL of the dermal bathing solution was removed at selected intervals (4, 8, 12, 16, 24 h) and analyzed. Each receptor sample was immediately replaced with an equal volume of fresh physiological solution. At 24 h the dermal bathing solution and the donor phase of each diffusion cell were recovered for the following analysis.

#### 2.5.2. Experiment 2

Experiment 1 was repeated using an abraded skin protocol as suggested by Bronaugh and Steward [21] skin was abraded by drawing the point of a 19-gauge hypodermic needle across the surface (20 marks in one direction and 20 perpendiculars). As donor solution was used 2.5 mL of Co_3_O_4_NPs suspension (606 μg·cm^−2^), dispersed in synthetic sweat at pH 4.5 to ensure an infinite dose.

#### 2.5.3. Blanks

For each experiment, two cells were added as blank. The blank cells were treated as the other cells with the exception that only synthetic sweat was used in the donor compartment.

#### 2.5.4. Skin Digestion after the Experiment

After the experiment, the skin pieces were washed three times with physiological solution to remove Co_3_O_4_NPs on the skin, then removed from the diffusion cells and treated as follows: skin samples from exp. 1 were separated into epidermis and dermis by heat shock, immerging in water at 60 °C for 1 min before freezing, while skin samples from exp. 2 were simply stored in a freezer at −25 °C. At the time of the analysis, the skin membranes were dried for 2 h at room temperature, then cut into sections, weighed and put into beakers with 10 mL of HNO_3_ 69% v/v and 2 mL of H_2_O_2_ for digestion. They were agitated for 24 h at room temperature than heated at the boiling point until the remaining solutions were of 2 mL in volume. The solutions were diluted to a volume of 10 mL with milliQ water for the analysis with ICP-AES.

### 2.6. Analytical Measurements

The metal content in the receiving phase and into the skin was analyzed by Inductively Coupled Plasma–Mass Spectrometry (ICP-MS) using an ELAN DRC II, (Perkin Elmer, Waltham, USA) instrument equipped with dynamic cell reaction (DRC). The calibration curve was prepared by dilution of standard solution ranging from 0.5 to 1000 µg/L (cobalt in HNO_3_ 2% mono elemental standard solution, Carlo Erba Reagenti, Milano, Italy). The calibration curve and sample solutions were pumped in the spray chamber using a peristaltic pump. The blank samples were used to correct for any contamination in each batch. The concentration of cobalt was expressed as microgram per liter. The accuracy of the method was determined on the basis of the mean values obtained on certified reference materials NIST 1643e-1643d trace elements in water (National Institute of Standards and Technology). The coefficients of variation ranged from 4% to 8% among series and from 6% to 12% between series and the limit of detection, calculated as three standard deviations of the background signal obtained on 10 blind samples, were 0.005 µg/L. The laboratory participates in the inter-comparison program for toxicological analysis in biological materials for the determination of cobalt (G-EQUAS of the German Society of Occupational and Environmental Medicine).

Total cobalt concentration in the donor phases and in the solutions resulting from the mineralization of the skin sample were performed by Inductively Coupled Plasma–Atomic Emission Spectroscopy (ICP-AES) using a Spectroflame Modula E optical plasma interface (OPI) instrument (by SPECTRO, Germany). The analysis were conducted using a calibration curve obtained by dilution (range: 0–10 mg/L) of Spectrascan^®^ cobalt standard solution for ICP-AES analyses (by Teknolab A/S, Norway). The limit of detection (LOD) at the operative wavelength of 228,616 nm was 0.05 mg/L. The precision of the measurements as relative standard deviation (RSD %) for the analysis was always less than 5%.

#### 2.6.1. Cell Tests

Stock solutions of Co_3_O_4_ (1 mg/mL ethanol) were diluted to the required concentrations (1.5 × 10^−7^–1.0 × 10^−3^ M, equivalent to 0.023–1500 μg/cm^2^) using the cell culture medium and sonicated before using.

#### 2.6.2. Cell Culture

Immortalized human keratinocyte cell line HaCaT [25] was purchased from Cell Line Service (DKFZ, Eppelheim, Germany). Cells were grown in Dulbecco’s Modified Eagle’s medium (DMEM) supplemented with 2 mM·L-Glutamine, 100 U/mL penicillin-100 µg/mL streptomycin and 10% fetal bovine serum (FBS). Cells were cultured in 75 cm^2^ cell culture flasks at 37 °C in a 5% CO_2_ atmosphere. All cell culture reagents were from Euroclone (Milan, Italy). Cells received fresh medium every 3 days and were subcultured every 7 days.

#### 2.6.3. MTT Assay

Cells (5 × 10^3^ cells/well) were plated in 96-wells plates for 24 h and then exposed to increasing concentrations of Co_3_O_4_NPs (1.5 × 10^−7^–1.0 × 10^−3^ M, equivalent to 0.023–1500 μg/cm^2^). After 24 h, 48 h and 7 days of exposure, cells were washed with PBS and a 10% MTT solution in complete medium was added a 10% MTT solution was added, and after 4 h the insoluble crystals were solubilized with DMSO [26]. To avoid artifacts in the optical density (OD) values, derived from the presence of particles, the solution was centrifuged for 2 minutes at 1300 rpm and transferred in a new plate. Plates were read in a Microplate Autoreader (Bio-Tek Instruments) at 540/630 nm. Data are reported as % of control and are the mean ± SE of 4 independent experiments performed in triplicate.

#### 2.6.4. AlamarBlue^®^ Assay

Cells (15 × 103 cells/well) were cultured in 96-wells plates. After 24 h, culture medium was removed and substituted with 200 µL of complete medium and cells exposed to different concentrations of Co_3_O_4_NPs (1.5 × 10^−7^–1.0 × 10^−3^ M, equivalent to 0.023–1500 μg/cm^2^). After 24 h, 48 h and 7 days, cells were washed to remove particles and a solution of 10% AlamarBlue^®^ in complete medium (final volume 200 μL) was added to each samples. After 4 h of incubation with the reagent in a humidified 5% CO_2_ atmosphere, the solution was carefully transferred in a black plate. Fluorescence intensity was read by a Fluorocount microplate Fluorometer (Packard, Germany) at an excitation wavelength of 530 nm and emission wavelength of 590 nm. Data are reported as % of control and are the mean ± SE of 4 independent experiments performed in triplicate.

#### 2.6.5. Propidium Iodide Uptake

Cells (5 × 103 cells/well) were seeded in 96-wells plates and after 24 h exposed to increasing concentrations of Co_3_O_4_NPs (1.5 × 10^−7^–1.0 × 10^−3^ M, equivalent to 0.023–1500 μg/cm^2^) for seven days. Propidium iodide (PI) uptake was performed as previously described [27,28]. Briefly, after treatment cells were washed 2 times with PBS and then rinsed with 200 μL of 3.0 × 10^−6^ M PI in PBS. After 30 min, fluorescence intensity was read by a Fluorocount microplate Fluorometer (Packard, Germany) with excitation length of 530 nm and emission length of 590 nm. Thereafter, all the samples were permeabilized with 1% Triton-X-100 for 30 minutes to obtain total cell content for each sample and fluorescence read. Positive control was obtained permeabilizing untreated cells with 1% Triton-X. Data are reported as % of positive control (equal to 100% PI uptake) after normalization on total cell content and are the mean ± SE of 3 independent experiments performed in triplicate.

### 2.7. Cell Fixation for TEM Analysis

HaCaT cells were seeded in cell culture dishes and when nearly to confluence, treated with 100 µM Co_3_O_4_NPs. After 24 h, cells were washed three times and fixed for 1 h in a solution of 2% glutaraldehyde (Serva, Heidelberg, Germany) in 0.1 M cacodylate buffer (pH 7.4). The fixed cells were washed twice (10 minutes each) with 0.1 M cacodylate buffer and then post-fixed with 1% osmium tetroxide for 1 h at 4 °C. Post-fixed samples were dehydrated with an ascending ethanol series ending with 100% ethanol and then embedded in Dow epoxy resin (DER332/732; Società Italiana Chimici, Rome, Italy). The last resin embedding was made under vacuum. Ultrathin sections were prepared with an Ultramicrotome Leica Ultracut UCT (Leica Microsystems, Milan, Italy) equipped with a diamond blade Drukker 3 mm (Emme3, Milan Italy). Ultra-thin sections were observed with a transmission electron microscope (EM208; Philips, Eindhoven, The Netherlands) and micrographs acquired with a Morada camera (Olympus Soft Imaging Solutions (OSIS), Munster, Germany). Double stain was not performed to avoid interference with NPs.

### 2.8. Statistical Analysis

Co concentration data (μg·cm^−3^) in the receptor solution were converted to the total amount that penetrated (μg·cm^−2^), with a correction for dilution due to sample removal.

Data analysis was performed with Excel for Windows, release 2007 and Stata Software, version 11.0 (StataCorp LP, College Station, TX, USA). Skin absorption data were reported as mean ± standard deviation (SD). The difference among independent data was assessed by means of the Mann-Whitney test.

Cytotoxicity data were reported as mean ± standard error (SE) of at least three independent experiments performed in triplicate. The concentration giving the 50% of the maximal effect (EC_50_) was calculated using the GraphPad software version 4.0 (Prism GraphPad, Inc.; San Diego, CA, USA).

## 3. Results

### 3.1. Nanoparticles Characterization

**Figure 1:**
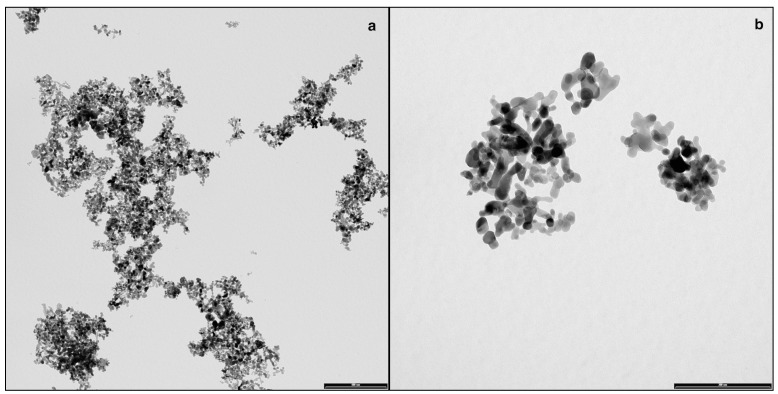
(**a**,**b**) Representative TEM (Transmission Electron Microscopy) images of agglomerated Co_3_O_4_NPs dispersed in synthetic sweat at the beginning of the experiments (bar: **a** = 500 nm, **b** = 200 nm).

**Figure 2:**
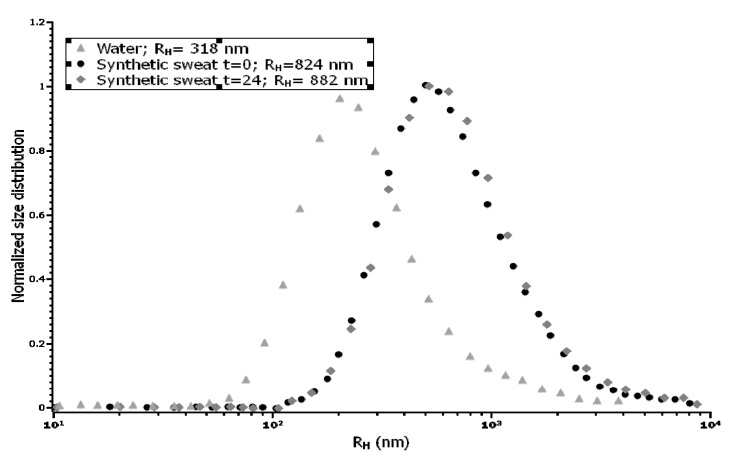
Size distribution of Co_3_O_4_NPs in water and in synthetic sweat suspension, estimated by DLS (Dynamic Light Scattering).

TEM characterization of cobalt-oxide NPs (Co_3_O_4_) specimens showed that NPs were irregular and not spherical, with a tendency to form agglomerates of some decades of NPs (Figure 1a,b). The size distribution of NPs was narrow and centered around a mean value of 17 ± 0.2 nm [29]. No differences in aggregation were found in donor solution at 0 and 24 h The hydrodynamic radius value (R_H_) observed in water was centered in 318 nm, while it changed considerably when assessed in synthetic sweat, reaching a value higher than 800 nm (Figure 2) and quite stable during all the time of the experiment (824 at t_0_ and 882 nm at t_24_). This phenomenon was clearly in agreement with the measured Z-potential values, reported in Table 1. The surface charge values suggested that Co_3_O_4_NPs were more stable in water, thanks to their higher electrostatic stabilization. Results derived from the ultrafiltration of the NPs suspension showed that the cobalt concentration was always less than 0.1% of the original NPs dispersion.

**Table 1:** Comparison of Z-potential values in water and in synthetic sweat.

Medium Specimen	Water	Synthetic Sweat T = 0	Synthetic Sweat T = 24 h
Co_3_O_4_	Mean: –19.8 +/− 1.15 mV	Mean: –18.5 +/− 3.5 mV	Mean: –15.9 +/− 4.2 mV

### 3.2. NPs Skin Permeation

**Figure 3:**
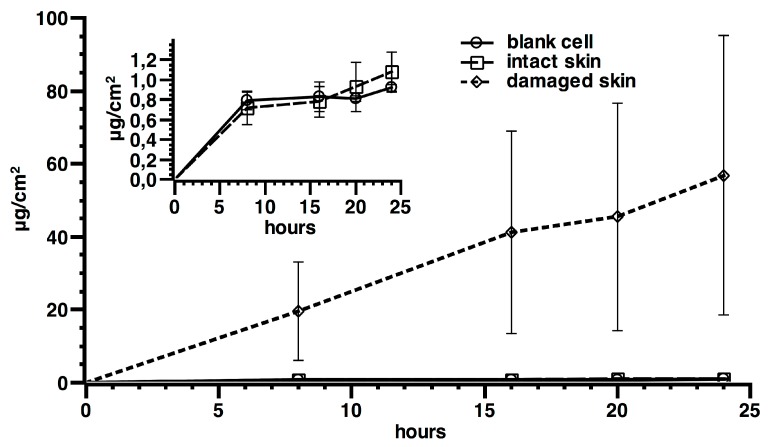
Cobalt permeation profile after skin application of Co_3_O_4_NPs on intact and damaged skin (main graph). Differences between intact skin, controls exposed to the ultra filtered solution, and blanks are reported in the small box (results expressed as means and standard deviation). Six replication for each experiment.

In experiments with intact skin and in blanks, the concentration of cobalt in receiving phases was similar without an increase of the cobalt concentration during time and so a permeation flux was not achievable (Figure 3). In experiment 2, where damaged skin was used, a metal permeation was found, with flux values of 2.1 ± 2.0 ng·cm^−2^·h^−1^ and a lag time of 4.3 ± 2.1 h (mean and standard deviation). The amount of cobalt permeated through skin in 24 h was significantly higher using the damaged skin protocol (57 ± 38 ng·cm^−2^), while no significant differences were shown in intact skin between blank cells (0.92 ± 0.03 ng·cm^−2^) and those exposed to Co_3_O_4_NPs (1.08 ± 0.20 ng·cm^−2^).

**Figure 4:**
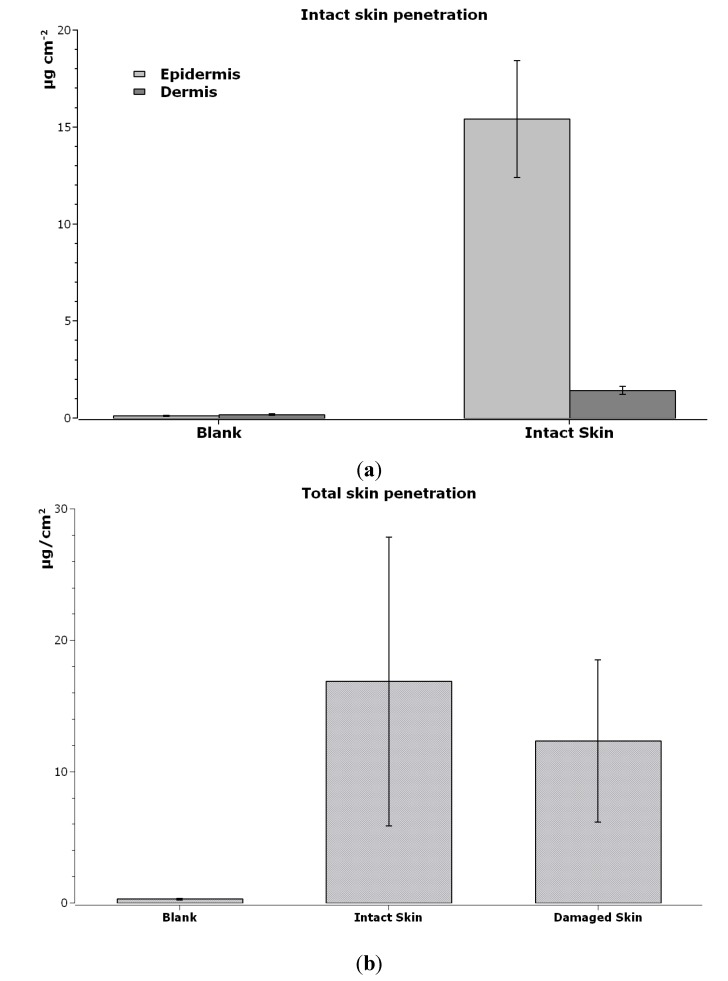
(**a**) Cobalt content (µg/cm^2^) inside each layer of intact skin, exposed to Co_3_O_4_NPs and only to physiological solution (blank cells). Mean and standard deviation of sixcells each. (**b**) Cobalt content (µg·cm ^−2^) inside the skin (epidermis + derma) of blank cells (exposed to physiological solution), intact skin and damaged skin (exposed to Co_3_O_4_NPs). Mean and standard deviation of six cells each.

ICP-AES skin analysis revealed a higher amount of cobalt in epidermis (15.43 ± 3.01 µg·cm^−2^) than in dermis (1.42 ± 0.21 µg·cm^−2^) in intact skin (exp. 1 *p* < 0.05, Figure 4a). Damaged skin had lower Co content than intact skin (12.31 ± 6.18 µg cm^−2^ *vs* 16.85 ± 10.98 µg cm^−2^, respectively), without reaching statistical significance (Figure 4b), suggesting that Co can be “stored” inside the skin.

### 3.3. Effect of Co_3_O_4_NPs on Cell Viability

**Figure 5:**
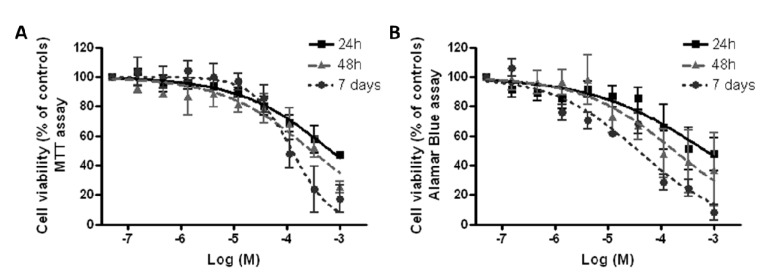
Cytotoxicity of Co_3_O_4_ NPS. Cell viability was measured by MTT assay (**A**) and Alamar Blue assay (**B**) after 24 h, 48 h and seven days exposure to Co_3_O_4_NPs (1.5 × 10^−7^–1.0 × 10^−3^ M, or 0.023–1500 μg/cm^2^) on HaCaT cells. Data are reported as % of untreated controls (equal to 100% cell viability) and are the mean ± SE of four independent experiments performed in triplicate.

Cytotoxicity of Co_3_O_4_NPs was evaluated on HaCaT cells using two different viability tests: the MTT assay, which is mainly an index of mitochondrial activity, and the AlamarBlue^®^ assay, which is an index of total cell viability. Cells were exposed to increasing concentrations of Co_3_O_4_NPs (1.5 × 10^−7^–1.0 × 10^−3^ M) for different times (24 h, 48 h and seven days). As shown in Figure 5, both cell viability assays, the MTT reduction assay (Figure 5A) and the AlamarBlue^®^ assay (Figure 5B), indicate that at the highest concentration (1.0 × 10^−3^ M), Co_3_O_4_NPs significantly reduced cell viability by 47.1% ± 1.6% and 47.6% ± 7.3% (MTT and AlamarBlue^®^ assays, respectively) after 24 h exposure and by 25.4% ± 3.9% and 37.3% ± 9.5% (MTT and AlamarBlue^®^ assays, respectively) after 48 h exposure. However, only after seven days exposure a concentration-dependent effect was evidenced so that EC_50_ values could be calculated and were equal to 1.3 × 10^−4^ M (95% confidence intervals, CI = 0.8–1.9 × 10^−4^ M, equal to 19.6 μg/cm^2^, CI 12.0–28.6 μg/cm^2^) and 3.7 × 10^−5^ M (95% CI = 2.2–6.1 × 10^−5^ M, equal to 5.57 μg/cm^2^, CI 3.31–9.18 μg/cm^2^), for the MTT and AlamarBlue^®^ assays, respectively.

### 3.4. Effect of Co_3_O_4_NPs on Plasma Membrane Damage

**Figure 6:**
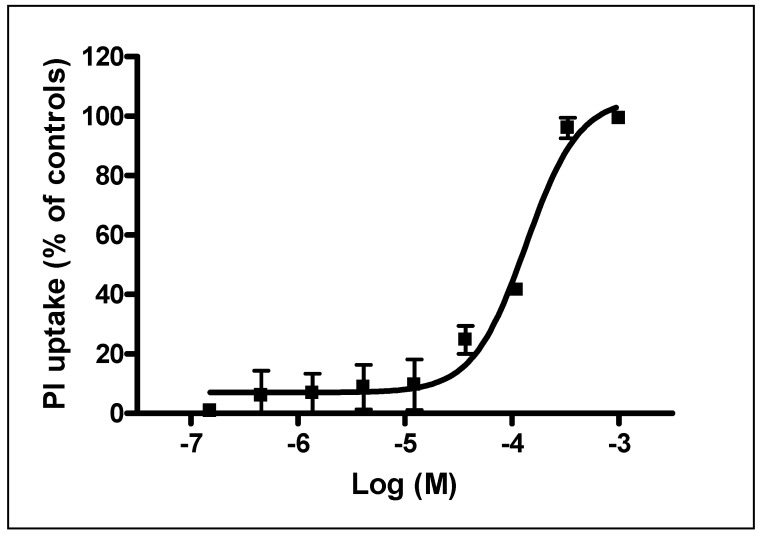
PI uptake in HaCaT cells exposed for seven days to Co_3_O_4_NPs (1.5 × 10^−7^–1.0 × 10^−3^ M, or 0.023–1500 μg/cm^2^). Data are reported as % of PI uptake with respect to positive control (Triton X-100, equal to 100% PI uptake) and are the mean ± SE of three independent experiments performed in triplicate.

To evaluate if cytotoxicity induced by Co_3_O_4_NPs was associated to plasma membrane damage, Propidium iodide (PI) uptake was evaluated. As shown in Figure 6, exposure to Co_3_O_4_NPs (1.5 × 10^−7^–1.0x10^−3^ M) for seven days induced a concentration-dependent increase of PI incorporation (99.3% ± 0.7%) that at the highest concentration (1.0 × 10^−3^ M) was comparable to that of the positive control, Triton-X-100 (100%). The calculated EC_50_ value was equal to 1.3 × 10^−4^ M (95% CL = 0.9–1.9 × 10^−4^ M, equal to 19.6 μg/cm^2^, CI 13.6–28.6 μg/cm^2^).

### 3.5. Evaluation of Cellular Internalization of NPs Using Electron Microscopy Imaging

**Figure 7:**
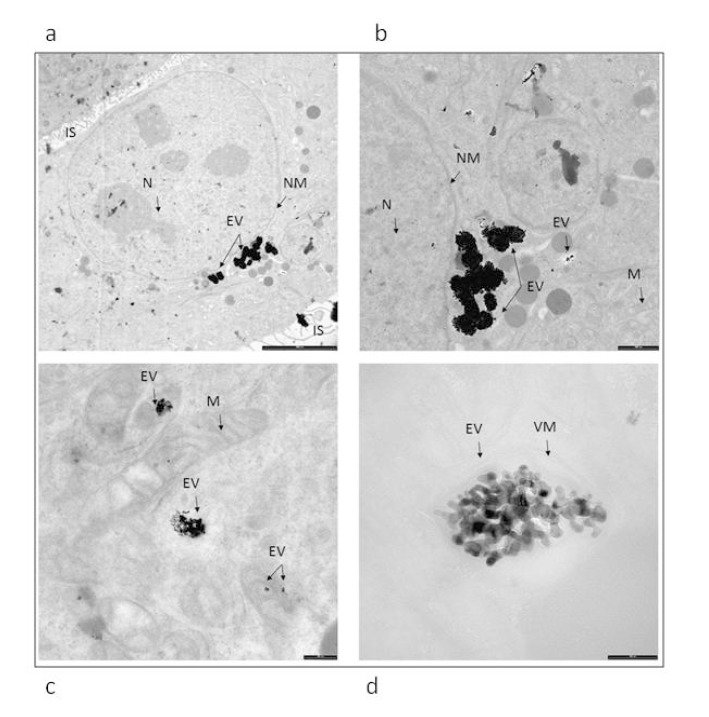
Ultrastructure of *in vitro* culturing keratinocytes exposed for 24 h to Co3O4NPs. ((**a**) bar 5um, (**b**) bar 200 nm, (**c**) bar 200 nm, (**d**) bar 100 nm). Electron-dense material of NPs aggregate is observed inside the organelles. EV: endocytic vesicles, IS: intercellular space, N: nucleus, NM: nuclear membrane, VM: vesicles membrane, M: mitochondria.

In Figure 7 it is possible to visualize electron-dense clusters of NPs aggregate inside the organelles. No NPs were detected inside the nucleus.

## 4. Discussion

For the first time, we studied skin absorption of cobalt-oxide NPs using an *in vitro* protocol on human skin. Our results add important information on knowledge on NPs interaction with human body and help us to understand the human risk related to NPs contamination. We demonstrated that cobalt oxide NPs can cross the skin, but only when this barrier is damaged. No absorption at all has been demonstrated through intact skin applying Co_3_O_4_NPs. No ions release was detected in donor solution.

It is known that metal NPs can penetrate (into the skin) and permeate (pass through the skin) as nanoparticles if they are very small (4 nm for Quantum dots [30]) or, more commonly, they can release a high percentage of ions, which eventually cross the skin barrier [20]. The dissolution of NPs is a relevant matter for material in nano-size range, since the high surface to volume ratio increases the risk of free metal ions release when compared to materials in traditional form [31].

For metal oxides, which are more stable and less-soluble than their metal counterpart [32], this release is negligible [33,34] and cobalt oxide NPs have been shown to be less toxic than cobalt ions [35] Nevertheless, at a cytological level, cobalt oxide NPs can release ions with a Trojan-horse type mechanism [32] and cause rapid induction of ROS [35], and with ROS levels higher than those induced by cobalt ions [15,29,36,37]. In angiogenic cells exposure to Co_3_O_4_NPs significantly reduced cell viability and increased pro-inflammatory cytokine gene expression [38].

To assess the penetration capability of the cobalt oxide NPs through the skin barrier, we compared the results of the present study with the ones obtained in a previous one, where metallic cobalt NPs have been tested using a similar protocol, but owned a larger size [17]. Table 2 shows that the metal content in damaged skin was similar when Co_3_O_4_NPs are used (89.6% respect to CoNPs exp), while metal concentration in receiving solution was significantly lower (5.6%) as well as flux through the skin (5%). From this point of view, considering also the smaller size of Co_3_O_4_NPs, it is possible to state that Co_3_O_4_NPs are safer than CoNPs, with regard to the permeation of the skin. When intact skin is used, only CoNPs can permeate the barrier and Co can be found in receiving phases, while no permeation at all was detectable after the application of Co_3_O_4_NPs. These differences can be explained by the fact that CoNPs can release cobalt ions [39], which can permeate easily the skin, while Co_3_O_4_NPs are very stable in physiological solution and cannot release ions [33,34], as demonstrated by scientific literature and confirmed in this study by the ultra filtration of the solution used as donor phases.

It can be concluded that when the skin barrier is damaged or affected by diseases that change barrier properties metal oxide absorption is feasible. This suggests the need for a better protection in people and workers with skin diseases exposed to metal and even to metal oxide NPs, as barrier disruption of the skin is common in workers and in atopic subjects [40]. Nevertheless, we demonstrated that our cobalt oxide nanoparticles could not permeate the normal skin, confirming that when metal NPs cannot release ions, the permeation is not so easy, as was demonstrated for CoNPs, which can release ions.

Comparison between cobalt oxide NPs, cobalt NPs and cobalt as bulk material [17] permits to understand better the potential that metal and metal oxide NPs present in relation to skin absorption. As CoNPs can permeate the skin in higher amount than bulk material, as previously demonstrated, our cobalt oxide NPs are stable and cobalt content in receiving phases is zero in intact skin and very low also in damaged skin.

**Table 2:** Co and Co_3_O_4_ concentration (µg/cm^2^) into the skin and in receiving solution after 24 h exposure. To compare values with a previous study [26], we standardized results considering the different concentration of Co and Co_3_O_4_ in donor solution.

**Damaged Skin**	**Donor Suspension**	**Co_3_O_4_NPs (Peak 17 nm) 606 μg·cm^−2^ (445 μg·cm^−2^ as Cobalt)**		**CoNPs (Peak 80 nm) 1000 μg·cm^−2^**		**Co_3_O_4_NPs Standardized Values 1000 μg·cm^−2^**	
		**Mean**	**SD**	**Mean**	**SD**	**Mean**	**%**
	Membrane (μg·cm^−2^)	4.78	0.90	12.0	3.8	10.75	89.6%
	Receiving Solution (ng·cm^−2^)	47	41	1870 *	860	106	5.6%
	Flux (ng·cm^−2^·h^−1^)	1.7	2.0	76 *	49.3	3.82	5.0%

* Mann-Whitney test *p* < 0.01.

Finally, the cytotoxic properties of Co_3_O_4_NPs were characterized on HaCaT cells, a human non-tumor keratinocyte cell line that is widely used as a simple model to assess cytotoxicity at the skin level [41]. Cytotoxicity was evaluated using a solution with NPs concentration similar to that used in permeation studies, performing two different assays: the MTT assay, that relies on the activity of mitochondrial dehydrogenases, and the AlamarBlue^®^ assay, that involves also cytoplasmatic dehydrogenases [42,43]. In the HaCaT model, both methods evidenced with a similar pattern the ability of Co_3_O_4_NPs to reduce cell viability. However, an exposure time as long as seven days was required to induce a concentration-dependent cytotoxic effect, whereas at shorter exposure times (*i.e.*, 24 or 48 h) a significant cytotoxic effect was observed only at the highest concentrations used. To better characterize Co_3_O_4_NPs-induced cytotoxicity, PI uptake was evaluated after seven days of exposure. Under this condition, Co_3_O_4_NPs caused a concentration-dependent PI incorporation, index of plasma membrane rupture. On the whole, these data demonstrated that Co_3_O_4_NPs are able to induce significant cytotoxic effects after a long time exposure (*i.e.*, seven days of exposure) and that this effect seems to be due to a damage at the plasma membrane level. These data, if confirmed on more complex models, could have a significant impact on the evaluation of the human risk associated to cutaneous exposure to these NPs.

## 5. Conclusions

Skin absorption of NPs is a matter of concern for workers and users that can be exposed to objects, powders and solution containing NPs. Our study demonstrated that Co_3_O_4_NPs cannot permeate through intact skin and that only a very low concentration of cobalt is detectable in receiving solutions when a damaged skin protocol is used. However, our results on cultured keratinocytes suggest that a long-term exposure to Co_3_O_4_NPs could induce cell damage and necrosis. We thus recommend the use of personal protective equipment to avoid contamination of the skin with NPs because the impaired skin barrier is common among workers and atopic subjects.
